# Spontaneous intracerebral hemorrhage during computed tomography scanning—assessment of hyperacute hematoma growth

**DOI:** 10.1007/s11357-025-01696-5

**Published:** 2025-05-10

**Authors:** Bence Gunda, Péter Böjti, Tímea Takács, Esra Zhubi, Dániel Bereczki, Andrea Varga, Lajos R. Kozák

**Affiliations:** 1 Department of Neurology, Semmelweis University, Balassa Utca 6, 1083 Budapest, Hungary; 2 Heart and Vascular Centre, Semmelweis University, Budapest, Hungary; 3 Department of Neuroradiology, Medical Imaging Centre, Semmelweis University, Budapest, Hungary

**Keywords:** Bleeding sources, CT angiography, cranial CT, Hematoma expansion, Hyperacute, Spontaneous intracerebral hemorrhage

## Abstract

Pathophysiological mechanisms underlying hematoma expansion in spontaneous intracerebral hemorrhage (ICH) remain poorly understood, and most data are derived from postmortem studies or serial neuroimaging studies performed over hours to days from onset. Our unique case report of a hypertensive ICH serendipitously captured by serial CT provides valuable in vivo data from the very onset of hematoma formation in an aging individual. A 76-year-old hypertensive man underwent elective carotid CT angiography to evaluate a previously known asymptomatic right carotid stenosis. During scanning, he developed severe right hemispheric neurological deficit signs. Immediate rescanning and subsequent follow-up imaging revealed the hyperacute evolution of a right putaminal ICH. We co-registered four scans (from 00 h:00 min, 00 h:06 min, 00 h:21 min, and 24 h:58 min) to a common template in 3D and made volumetric measurements of the growing hematoma also assessing the spatial relationship of expansion with the sources of bleed seen as contrast extravasation (“spot signs”). We found that spot signs appeared on the periphery of the initial hematoma, and further expansion was seen in the directions determined by these spot signs. Most of the final ICH volume developed in the first 20 min post-onset, highlighting the hyperacute nature of hematoma growth. Our findings support the hypothesis that hematoma expansion in hypertensive ICH, particularly in aging individuals, results from multiple sources of bleeding due to a cascade of secondary vessel ruptures with eccentric expansion rather than a single source and continuous bleeding with concentric expansion reflecting the global fragility of the cerebral vasculature. The therapeutic time window for hematoma expansion prevention is very narrow.

**Supplementary Information:**

The online version contains supplementary material available at 10.1007/s11357-025-01696-5.

## Introduction

Despite recent advances in medical therapy, spontaneous intracerebral hemorrhage (ICH) remains a devastating neurological condition with limited therapeutic options where the main goal of treatment is the prevention of further hematoma growth usually occurring in the initial hours after onset [1]. However, pathophysiological mechanisms underlying hematoma expansion are still poorly understood [1].

Most data come from postmortem studies that describe two possible mechanisms: a single central vessel rupture and continuous bleeding with concentric expansion, or an initial bleed starting a tissue shockwave causing a cascade of secondary vessel ruptures with eccentric expansion [2]. In vivo evidence is limited and largely derived from serial neuroimaging studies typically performed hours to days from onset [3, 4]. Notably, one study reported that hemorrhages tend to expand non-uniformly around their surface, challenging the model of concentric growth [5]. Similarly, catheter angiography within 1 h of the onset of a hypertensive ICH revealed active bleeding from multiple lenticulostriate arteries, supporting the cascadic model of expansion [6]. Bleeding onset and hyperacute evolution within minutes have only rarely been captured with neuroimaging [7, 8]. One reported case of ICH occurring during magnetic resonance imaging (MRI) in a patient with probable cerebral amyloid angiopathy showed asymmetric, non-uniform expansion, further supporting the hypothesis of secondary vessel ruptures in a cascading pattern [9].

In this context, we present a unique case of hypertensive ICH serendipitously captured by serial computed tomography (CT) and CT angiography (CTA) from the very onset. This report provides critical in vivo data on the hyperacute pattern of hematoma evolution and its spatial relationship to active bleeding sources. This case was previously published as an “Images in Neurology” in JAMA Neurology [8]; the present manuscript provides a detailed clinical context, a comprehensive follow-up, a labor-intensive post-processing and analysis of images and a discussion on ICH pathophysiology with a specific focus on aging-related mechanisms.

## Materials and methods

### Case presentation

A 76-year-old hypertensive male taking acetylsalicylic acid underwent elective carotid CTA for evaluation of a previously known asymptomatic right carotid stenosis. Prior to the examination, the patient had no neurological symptoms, was independently ambulating, and was able to position himself on the scanner table. During the scanning process, he developed an acute rightward head and gaze deviation. Immediate clinical evaluation revealed left-sided hemiplegia, hemisensory loss, hemianopia, neglect, and severe eye and head deviation to the right. An urgent repeat CT scan was conducted. The images demonstrated an expanding right putaminal intracerebral hematoma with multiple points of contrast extravasation.

The patient’s coagulation parameters were normal, but his blood pressure was markedly elevated (190 mmHg systolic). He was admitted to the intensive care unit, intubated due to decreased level of consciousness, and managed with antihypertensive therapy, osmotic agents, and comprehensive supportive care. Follow-up imaging the next day showed further hematoma expansion. Later scans showed stabilization and resolution of the hematoma without any underlying neoplastic or macrovascular lesion. The patient eventually died due to complications 70 days after his stroke.

### Imaging and post-processing

**Fig. 1:**
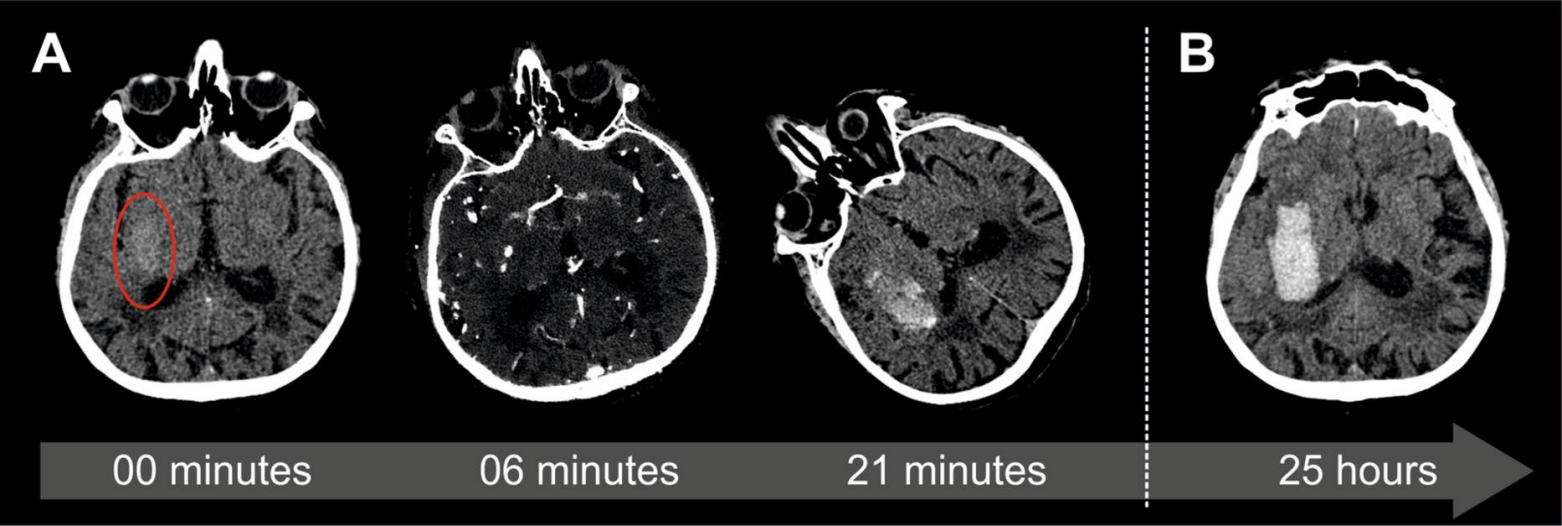
Hyperacute right putaminal hemorrhage on consecutive CT scans. **A** Hyperacute phase: non-contrast CT (0 min), CTA (6 min), and CT (21 min). **B** Acute phase: non-contrast CT (25 h). The red circle shows an area of slight hyperdensity representing fluid blood. Acute neurological deterioration demonstrated by progressive head and eye deviation (panel **A**) on image planes containing at least one of the lenses. The slice shown in panel **B** is 16 mm above the plane of the lenses

**Fig. 2:**
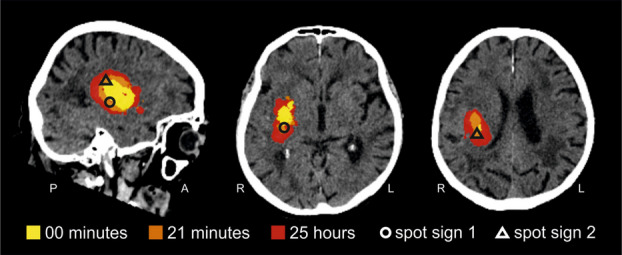
Color-coded hematoma expansion. Color-coded stepwise hematoma expansion in one sagittal and two axial planes (at the levels of spot signs) with the indication of spot signs: yellow: 0 min, orange additional hemorrhage at 21 min; red: additional hemorrhage at 25 h; markers: spot signs

The first non-contrast CT of brain was performed at 13:58, followed by CTA at 14:04. A repeat CT was performed at 14:19 after noting the patient’s symptoms, and a follow-up CT was conducted the next day at 14:56 (Fig. 1). These four scans (from 00 min, 06 min, 21 min, and 24 h:58 min) were co-registered to the space of the original non-contrast CT examination to assess the spatial relationship of expansion with the sources of bleed seen as contrast extravasation on CTA using SPM12 (https://www.fil.ion.ucl.ac.uk/spm/). The data are visualized as overlays on the 25-h follow-up NCCT (Fig. 2, Supplemental Video). The extent of hemorrhages was traced on the co-registered image volumes in MRIcron (https://www.nitrc.org/projects/mricron) using 3D region growing based on density (HU) information.

## Results

The first non-contrast CT (NCCT) of brain at 0 min showed a very vague but homogenous hyperdensity measuring 30 × 15 × 21 mm (4.73 ccm) in the right putaminal region, with an attenuation of 40 Hounsfield Units (HU) (Fig. 1A 0 min; yellow region in Fig. 2). At 6 min, CTA demonstrated two small eccentric areas of contrast extravasation (spot signs) above and below the original hyperdensity (Fig. 1A 6 min; markers in Fig. 2). No vascular malformation or aneurysm was detected.

By 21 min, repeat brain CT revealed significant hematoma expansion, now measuring 46 × 25 × 33 mm (18.98 ccm), with heterogeneous hyperdensity ranging from 40 to 70 HU and associated mass effect (Fig. 1A, 21 min; orange region in Fig. 2). Neurological deterioration simultaneous to hyperacute hematoma expansion could be appreciated by the progression of eye and head deviation (Fig. 1A). A follow-up scan at 24 h and 58 min showed further hematoma extension, increasing to 50 × 25 × 42 mm (24.15 ccm) (Fig. 1B; red region in Fig. 2).

## Discussion

To the best of our knowledge, this case demonstrates for the first time the earliest possible presentation and evolution of a spontaneous hypertensive ICH alongside its sources of bleeding, captured serendipitously by CT and CTA, thereby providing invaluable in vivo data on the pathomechanism of ICH.

Unlike the hyperdensity of 60–70 Hounsfield Units (HU) normally observed in an acute ICH within a few hours of onset, which represents clotted blood, our first scan obtained during or immediately after onset presented a density of 40 HU, consistent with circulating blood (Fig. 1A 0 min) [10]. On CTA 6 min later, two spot signs appeared immediately above and below the initially seen hematoma (Figs. 1A and 2). On the second brain CT 21 min post-onset (Fig. 1A), the appearance of the homogenous 40 HU initial bleed remained largely unchanged but was surrounded by contrast containing a new bleed of 60–110 HU with two foci that developed around the spot signs and on the periphery of the initial hematoma (Fig. 2; Supplementary Video). Further hematoma expansion in the directions determined by the spot signs was seen on the third brain CT 1 day later (Figs. 1B and 2; Supplementary Video), confirming the predictive role of the spot sign [11].

Recent evidence links hematoma heterogeneity on non-contrast CT, such as swirl, blend, and black hole signs, to hematoma expansion [12]. The extreme heterogeneous density seen on CT at 21 min in this case supports this association. However, in our case, this is not a truly non-contrast scan as it also shows previous contrast extravasation, adding to the heterogeneity. The observed spatial pattern of non-uniform hematoma extension towards directions determined by the sources of active bleeding on the periphery of an initial bleed strongly supports the pathophysiological hypothesis of multiple sources of bleed due to a cascade of secondary vessel ruptures with eccentric expansion, rather than a single source and continuous bleeding with concentric expansion [2].

These findings align with previous reports on MRI, capturing cerebral amyloid angiopathy-related ICH showing asymmetric, non-uniform expansion [9], and catheter angiography in hyperacute hypertensive ICH showing active bleeding from multiple lenticulostriate arteries [6]. Therefore, there is growing evidence that the prevention of secondary vessel ruptures may be a therapeutic target to prevent hematoma expansion, the most devastating complication of ICH [13]. Our findings further emphasize the narrow therapeutic time window for hematoma expansion prevention as most of the final hematoma volume in this case developed within the first 20 min.

**Fig. 3:**
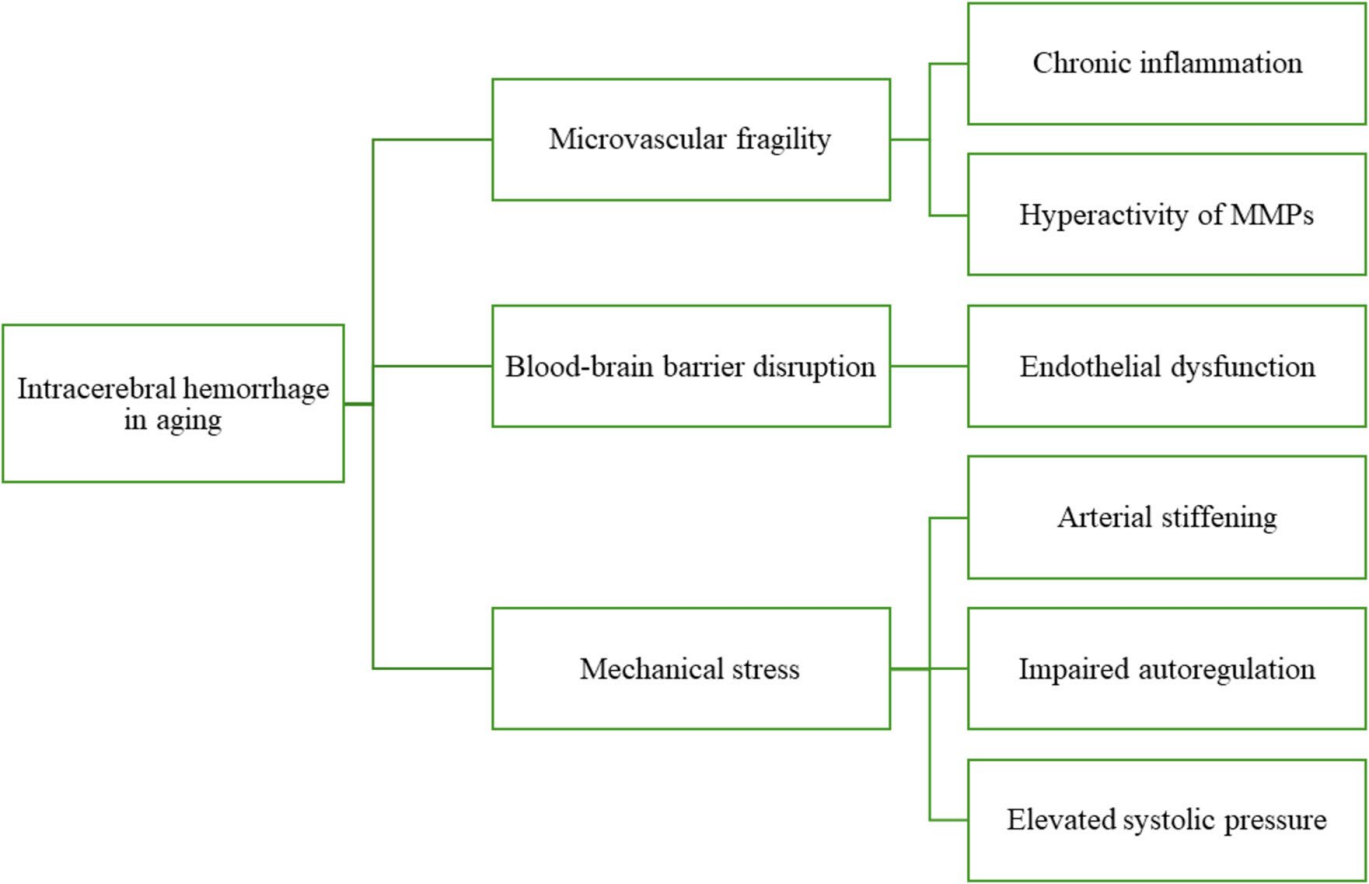
Pathomechanisms of intracerebral hemorrhage in aging individuals

The increased susceptibility to ICH in the elderly is due to a combination of age-related processes including endothelial dysfunction (“endothelial senescence”), chronic inflammation (“inflammaging”), arterial stiffening, and impaired autoregulation (as shown in Fig. 3) [14]. Endothelial senescence plays a key role, with aging endothelial cells showing reduced nitric oxide (NO) bioavailability, increased oxidative stress, and elevated pro-inflammatory signaling [15]. These senescent endothelial cells contribute to loss of vascular homeostasis, increased permeability, and breakdown of the blood–brain barrier (BBB), thus creating a permissive environment for hemorrhage [16]. Critically, senescence-associated secretory phenotype (SASP) involves secretion of cytokines, chemokines, and extracellular matrix-degrading enzymes, including matrix metalloproteinases (MMPs), which drive a state of chronic low-grade inflammation known as inflammaging, which exacerbates vascular injury and increases the risk of vessel rupture [17].

Moreover, the age-related decline in anti-geronic factors such as insulin-like growth factor 1 (IGF-1) undermines key protective mechanisms against microvascular senescence [18, 19], increases the production of reactive oxygen species (ROS), and dysregulates matrix metalloproteinase (MMP) activity, thereby contributing to vascular fragility and heightened susceptibility to intracerebral hemorrhage [20]. Hyperactivity of matrix metalloproteinases (MMPs), especially MMP-2 and MMP-9, results in extracellular matrix degradation and breakdown of the blood–brain barrier [21].

Age-related arterial stiffening and increased pulse wave velocity amplify central systolic pressure and mechanical stress on small cerebral vessels, while impaired autoregulation limits their ability to constrict, together heightening the risk of vessel rupture under elevated perfusion pressure [14]. In the setting of chronic hypertension, the rise in pulse wave velocity with aging leads to elevated systolic pressures being centrally transmitted to vulnerable compact intracerebral arterioles, further increasing the risk for vessel rupture [22]. Moreover, dysregulation of the renin-angiotensin system in aging also plays a role in cerebrovascular damage by promoting endothelial dysfunction and vascular remodeling, ultimately contributing to an increased risk of ICH [23]. The global vessel fragility due to the aforementioned mechanisms in our elderly hypertensive patient is well reflected by the observed cascadic hematoma expansion.

## Conclusion

Our findings support the hypothesis that hematoma expansion in hypertensive ICH results from multiple sources of bleeding due to a cascade of secondary vessel ruptures with eccentric expansion reflecting the global fragility of the cerebral vasculature. The therapeutic time window for hematoma expansion prevention is very narrow.

## Supplementary Information

Below is the link to the electronic supplementary material.

Supplementary file1 (MP4 8018 kb)

## Data Availability

Data are available on request from the corresponding author.
